# Comparative Analysis of Genomic and Pedigree-Based Approaches for Genetic Evaluation of Morphological Traits in Pura Raza Española Horses

**DOI:** 10.3390/genes16020131

**Published:** 2025-01-23

**Authors:** Chiraz Ziadi, Sebastián Demyda-Peyrás, Mercedes Valera, Davinia Perdomo-González, Nora Laseca, Arancha Rodríguez-Sainz de los Terreros, Ana Encina, Pedro Azor, Antonio Molina

**Affiliations:** 1 Departamento de Genética, Universidad de Córdoba, 14014 Córdoba, Spain; z72zizic@uco.es (C.Z.); ge1moala@uco.es (A.M.); 2 Departamento de Agronomía, ETSIA, Universidad de Sevilla, 41013 Sevilla, Spain; mvalera@us.es (M.V.); nlaseca@us.es (N.L.); 3 Departamento de Producción Animal, Universidad Complutense de Madrid, 28040 Madrid, Spain; daperdom@ucm.es; 4 Real Asociación Nacional de Criadores de Caballos de Pura Raza Española (ANCCE), 41014 Sevilla, Spain; arancha@lgancce.com (A.R.-S.d.l.T.); anaencmar@gmail.com (A.E.); pedroazor@lgancce.com (P.A.)

**Keywords:** SNP-genotyping, single-step GREML, estimated breeding value, reliability, horses

## Abstract

Background: The single-step best linear unbiased predictor (ssGBLUP) has emerged as a reference method for genomic selection in recent years due to its advantages over traditional approaches. Although its application in horses remains limited, ssGBLUP has demonstrated the potential to improve the reliability of estimated breeding values in livestock species. This study aimed to assess the impact of incorporating genomic data using single-step restricted maximum likelihood (ssGREML) on reliability (R^2^) in the Pura Raza Española (PRE) horse breed, compared to traditional pedigree-based REML. Methods: The analysis involved 14 morphological traits from 7152 animals, including 2916 genotyped individuals. Genetic parameters were estimated using a multivariate model. Results: Results showed that heritability estimates were similar between the two approaches, ranging from 0.08 to 0.76. However, a significant increase in reliability (R^2^) was observed for ssGREML compared to REML across all morphological traits, with overall gains ranging from 1.56% to 13.30% depending on the trait evaluated. R^2^ ranged from 6.93% to 22.70% in genotyped animals, significantly lower in non-genotyped animals (0.82% to 12.37%). Interestingly, individuals with low R^2^ values in REML demonstrated the largest R^2^ gains in ssGREML. Additionally, this improvement was much greater (5.96% to 19.25%) when only considering stallions with less than 40 controlled foals. Conclusions: Hereby, we demonstrated that the application of genomic selection can contribute to improving the reliability of mating decisions in a large horse breeding program such as the PRE breed.

## 1. Introduction

The Pura Raza Española horse (PRE) is a native Spanish equine breed officially recognized since the establishment of its studbook in 1912. The active total PRE population, 282,066 horses, is mainly located in Spain but also distributed across 67 other countries [1]. The PRE is the main equine breed in Spain, representing approximately 70% of all registered purebred equids. The PRE breeding program, handled by the Royal National Association of Spanish Horse Breeders (ANCCE), has been focused on improving the breed’s conformation, functionality, and reproductive traits while also aiming to reduce inbreeding and preserve its genetic heritage [2]. To ensure the reliability of PRE studbook records, various molecular tools, including blood group analysis, biochemical polymorphisms, and microsatellite markers, have been employed for paternity verification since the early 1980s. These efforts have resulted in over 40 years of verified parental data, providing an accurate and reliable basis for genetic improvement efforts. This has enabled the development of an official breeding program that evaluates over 40 traits using a pedigree-based restricted maximum likelihood methodology (REML).

Morphological traits are very important in the breed as they provide the foundation for the functionality required in dressage competitions, the main use of PRE horses [3], to the point that conformation plays a key role in determining the horse’s economic value. Additionally, the PRE horse population is monitored for various defects associated with morphology, including cresty neck, a disease that reduces the market value of horses during sales and is considered a disqualifying morphological defect in the studbook for high scores [4]. For these reasons, obtaining accurate genetic parameters and ensuring the high reliability of estimated breeding values is crucial for the success of genetic improvement programs.

Historically, estimated breeding values in animal populations have been derived from phenotype and pedigree information, with the extensive literature available on the subject [5]. Nowadays, the development of genomic-based estimates is expected to offer greater accuracy, as they are not dependent on the quality and completeness of the pedigree. These methods allow for a precise assessment of the degree of similarity in the genetic markers of the animals under evaluation, providing a more direct measure of their genetic potential. The genomic selection was first proposed 25 years ago by Meuwissen et al. [6]. However, it was more recently, about 15 years ago, when advances in genotyping methods and bioinformatics procedures enabled the implementation of routine genomic evaluations and selection in livestock species. This was further facilitated by the development of the single-step genomic best linear unbiased prediction (ssGBLUP) approach for estimating genomic breeding values [7,8]. Using this method, all available information from both genotyped and non-genotyped relatives can be simultaneously employed in the analysis along with phenotypes [9], thereby improving the accuracy of breeding values’ estimation [10].

ssGBLUP is increasingly used in most livestock species, namely dairy cattle [7,11,12,13,14], dairy goats [15,16,17], dairy sheep [18,19,20], and pigs [21,22,23]. However, studies on horses remain scarce [24,25,26,27]. In this species, genetic evaluations are still primarily based on pedigree-based methods in most breeds where breeding programs are currently being developed. It has already been demonstrated that genomic selection strategies contribute to a significant reduction in generation intervals in horse breeding programs [24,28,29].

The aim of this study was to estimate genetic parameters and compare the reliabilities of the estimated breeding values for morphological traits between the traditional REML method and the single-step genomic REML method in the PRE horse breed.

## 2. Materials and Methods

### 2.1. Pedigree and Phenotypic Data

The PRE genealogical information and phenotypic dataset used in this study were provided by the Royal National Association of Spanish Horse Breeders (ANCCE). Morphological data consisted of 7152 records of eleven zoometric, two linear traits, and a defect trait collected from 2008 to 2022 (1 record per trait per horse, both males and females). The following zoometric traits were evaluated: height at withers (HW), scapular-ischial length (SiL), length of the shoulder (LS), dorsal-sternal diameter (DsD), length of back (LB), width of croup (WC), length of croup (LC), thoracic perimeter (TP), perimeter of anterior cannon bone (PACB), angle of the shoulder (AS), and angle of croup (AC). Linear traits evaluated included two morphological traits: lateral hock angle (LHA) and direction of hock rear view (DHRV). Additionally, the cresty neck (CN) was analyzed as a morphological defect. The zoometric traits were systematically collected in official breed controls by trained morphological evaluators using standard measuring sticks and non-elastic measuring tapes, as described by Sánchez-Guerrero et al. [3]. The linear traits were collected using the linear scoring system in PRE horses as detailed by Sánchez et al. [30]. The cresty neck trait was measured on a linearized scale, including 9 scores from 1 to 9, using an adaptation of the scoring system proposed by Carter et al. [31].

The pedigree was traced back to seven complete generations and nine equivalent generations, making a total of 41,888 animals.

### 2.2. Genotyping and Quality Control

Sample selection was performed aiming to capture the maximum variability of the population and to include the most representative horses of the population, with a low average relatedness among individuals. A total of 2916 PRE horses (928 males and 1988 females), belonging to 882 studs, were selected for genotyping. The genomic DNA of the horses was obtained from the ANCCE DNA bank.

These horses were genotyped using MD microarray comprising 71,590 SNPs evenly distributed across the whole genome.

The raw genotypes (final reports) were transformed into binary files (bed, bim, fam) using the R statistical environment V4.4.2 [32] and filtered using PLINK software v1.9 [33]. Markers with unknown chromosomes or positions within the chromosome, SNPs located on sex chromosomes, and those with a call rate below 95% or with a minor allele frequency below 0.01 were eliminated. As a result, the final genomic dataset included 61,271 SNPs.

### 2.3. Statistical and Genetic Analysis

The morphological traits were analyzed using the following multivariate model:

y=Xb+Za+e

where y is the vector of observations; b is the vector of fixed effects, including age (three classes: less than 4 years, between 4 and 7 years, and more than 7 years), sex (two classes: male and female), genetic origin (four classes: gray, bay, black, and chestnut), and geographical area of the stud (six classes: Spain, the rest of Europe, the United States and Canada, South America, Africa, and the Arabian Peninsula, and the rest of the world); a is the random additive genetic effect, and e is the random residual effect. X and Z are incidence matrices relating observations to fixed and random additive genetic effects, respectively.

The significance of the fixed effects for morphological traits was determined using the ‘GLM2’ R package [34]. All fixed effects were found to have a significant effect at a 0.05 significance level.

For all traits, the additive genetic effect uses two additive genetic relationship matrices: the A matrix, which denotes the pedigree-based additive genetic relationship for the traditional evaluation (REML), and the H matrix, which includes both pedigree and genomic relationships for ssGREML. The H matrix was derived by integrating the A matrix with the genomic relationship matrix G, calculated using the method proposed by VanRaden [35] as follows:

$$G=0.95\frac{{\mathrm{SS}}^{\prime }}{2\sum_{i=1}^{n}p_{i}\left(1-p_{i}\right)}+0.05A$$

where n is the number of SNP markers, $p_{i}$ is the allele frequency of marker i, A is the pedigree relationship matrix, and S is a centered incidence matrix of SNP markers.

Variance components estimated breeding values (EBVs) and genomic estimated breeding values (GEBVs) were estimated using restricted maximum likelihood (REML) and ssGREML approaches for classical and genomic evaluations, respectively. All analyses were performed using the HIBLUP v1.3.1 software [36].

## 3. Results and Discussion

This study has addressed, for the first time, a comprehensive genomic evaluation of a Spanish horse breed using the single-step genomic approach with a combined relationship matrix. By focusing on a diverse set of morphological traits, this research highlighted the potential of genomic tools to enhance breeding programs and demonstrated the improvement in efficiency and precision of large-scale equine genetic evaluation programs through the integration of genomic data.

### 3.1. Phenotypic Values

Descriptive statistics for the morphological traits analyzed in the PRE breed are presented in Table 1. The mean values for the zoometric traits ranged from 20.46 ± 1.26 cm for PACB to 191.59 ± 9.34 cm for TP, while the mean values for the linear traits were 5.14 ± 0.95 for LHA and 4.22 ± 0.91 for DHRV. The average mean of CN was 1.32 ± 0.84. The coefficients of variation (CV) for most zoometric traits in this study were low, except for AC (27.10%), whereas the linear traits showed higher CVs (18.4% for LHA and 21.53% for DHRV). CN presented the highest CV at 63.58% of all the traits studied. Average values per trait were similar to those reported by Poyato-Bonilla et al. [37] in a recent study on the PRE breed. In contrast, Sánchez-Guerrero et al. [3] analyzed the evolution of morphology in PRE mares and stallions over three time periods: animals born before 1990, those born between 1990 and 2001, and those born between 2002 and 2013. Their findings indicated lower values for HW (154.7 cm, 156.1 cm, and 157.9 cm, respectively) compared to the results observed in our study. However, they reported similar values for DsD (72.2 cm, 73.6 cm, and 73.9 cm), TP (190.0 cm, 192.1 cm, and 190.9 cm), and PACB (19.6 cm, 19.7 cm, and 20.2 cm) across the same periods. Our mean CN was lower than the values observed by Sánchez et al. [38] and Poyato-Bonilla et al. [39] in the same breed.

### 3.2. Genetic Parameters and Heritabilities Obtained Using REML and ssGREML

Table 2 shows the estimates of variance components and heritabilities for morphological traits derived from both REML and ssGREML approaches. The heritability estimates were similar using the A (REML) or the H (ssGREML) matrices, ranging from 0.08 (SE = 0.02) for CN to 0.76 (SE = 0.038) for HW. In contrast, LB and DHRV showed slightly higher $h^{2}$ estimates in the REML analysis.

The $h^{2}$ values reported by Poyato-Bonilla et al. [37] in PRE were significantly lower than ours (0.28 ± 0.04 for HW, 0.25 ± 0.01 for SiL, 0.19 ± 0.02 for LS, 0.14 ± 0.02 for DsD, 0.21 ± 0.01 for TP, and 0.17 ± 0.03 for PACB). Nevertheless, all these estimates were made without including genomic information. Our $h^{2}$ for CN was similar to the value estimated by Poyato-Bonilla et al. [39], but clearly lower than the 0.37 ± 0.03 value by Sánchez et al. [38].

The variations in estimates within the PRE breed may be attributed to differences in the models implemented, quality of data, pedigree recording, the number of available phenotypes, and the connectedness of the data [40]. These factors are all recognized to influence the estimation of genetic parameters.

Comparisons with other studies are difficult due to the limited number of genomic evaluations of horse morphology in the literature, with most studies focusing on only a few traits. Using a single-step approach, Vosgerau et al. [26] reported $h^{2}$ of 0.31 ± 0.08 for HW in German Warmblood horses, which was lower than the $h^{2}$ estimate obtained in our study. Similarly, Ricard et al. [27] observed $h^{2}$ values ranging between 0.14 (0.05) and 0.42 (0.07) for new morphological phenotypes obtained by linear combinations of Procrustes coordinates in French jumping horses.

Morphological traits are highly significant in the PRE breed due to their strong relationship with functionality, as these horses are primarily used in dressage competitions. Since most morphological traits have shown medium to high heritability, there is a great interest in obtaining highly reliable assessments as quickly as possible, as they can lead to a substantial genetic gain in the breed in short periods. Furthermore, with the increasing availability of genotypes in the PRE breed, the integration of genomic information into routine genetic evaluations will become possible.

### 3.3. Comparison Between the Reliability Obtained Using REML and ssGREML

The reliabilities of the estimated breeding values (EBVs) and genomic estimated breeding values (GEBVs) obtained using the REML and ssGREML methods for morphological traits are presented in Table 3. Results showed that the reliabilities of GEBVs derived from the ssGREML method were higher than those of EBVs obtained using the REML method.

Reliabilities ranged from 0.176 for CN to 0.298 for HW using REML and from 0.185 for CN to 0.327 for HW using ssGREML. Furthermore, when expressed as a percentage of differences, the overall increases in $R^{2}$ (considering all the animals in the pedigree) were in the range of 1.56% (DHRV) to 13.30% (AC).

Additionally, reliabilities obtained from the REML and ssGREML approaches were compared based on several criteria, including sex, the number of foals per sire, genotyped and non-genotyped animals, and the magnitude of the initial reliability obtained with REML. The results are provided in Table 4. The increase in reliability was similar for mares and stallions, except for SiL, LB, AS, AC, CN, and DHRV. Remarkably, the gain was greater in genotyped animals (6.9% to 22.7%) compared to non-genotyped animals (0.82% to 12.4%) and in stallions with fewer than 40 controlled foals (5.96% to 19.2%). Moreover, animals with previously low REML reliability demonstrated a significantly greater gain compared to those with higher initial reliability. This result could be attributed to the genomic information, which provides a more significant contribution by refining pedigree relationships (incorporates real kinship coefficient values), strengthening connections among individuals compared to BLUP, capturing additional genetic effects, and reducing residual variance [41]. Thus, substantially improving the accuracy of their genetic evaluation, where phenotypic and pedigree data were previously scarce.

**Figure 1:**
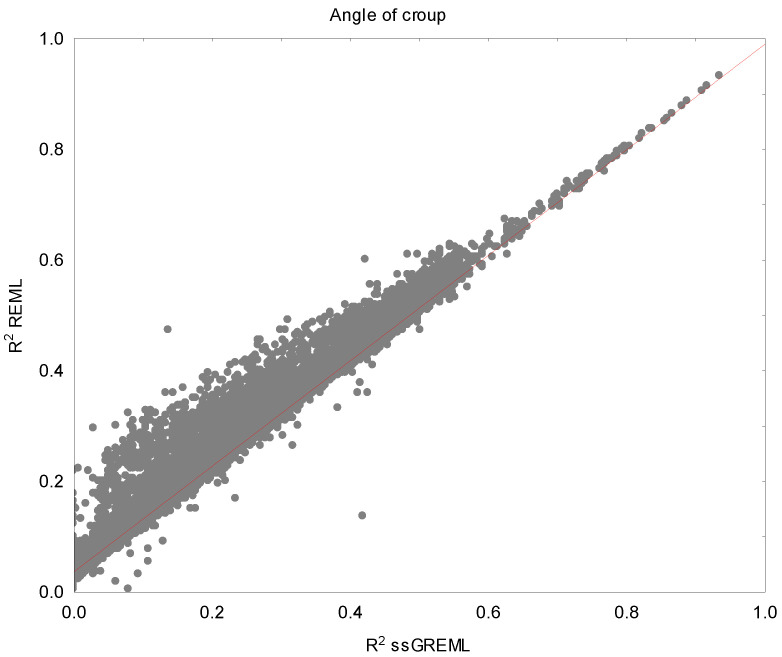
Comparison between the reliability obtained from REML ($R^{2}$ REML) and single-step GREML ($R^{2}$ ssGREML) for the angle of croup in the Pura Raza Española horse breed.

Figure 1 illustrates the comparison between the reliability obtained from REML ($R^{2}$ REML) and single-step GREML ($R^{2}$ ssGREML) of the angle of croup, the trait showing the greatest overall increase in reliability. It could be noticed that for animals with low REML reliability, the gain in reliability is larger.

The overall trend in the $R^{2}$ for morphological traits was unchanged as a function of heritability, likely because most of these traits already present medium to high heritability. On the contrary, in our previous study on comparing reliabilities for reproductive traits between REML and ssGREML methods in the PRE breed, the gain in $R^{2}$ increased as the heritability of the trait decreased, due to their low estimated heritability [42]. This finding aligns with the results reported by VanRaden et al. [43] and Misztal et al. [44]. ssGBLUP has been shown to outperform traditional methods in predicting traits with low heritability [45], such as those related to reproduction.

The results obtained in our study align with previous reports in livestock species where genomic evaluations are commercially available. In dairy cattle, the first species to integrate genomic evaluations at a commercial scale using the ssGBLUP approach, previous studies have demonstrated that ssGBLUP yielded higher accuracy compared to the traditional pedigree-based BLUP method, which relies only on pedigree information [7,11,46]. In dairy sheep, single-step evaluation has been shown to result in a 46.8% increase in accuracy compared to pedigree BLUP [18]. On the contrary, reports in horses are much scarcer, limited to a few traits and small populations. Vosgerau et al. [26] reported an average reliability of 0.35 using classical BLUP and 0.38 with ssGBLUP for the withers height trait in German Warmblood horses, resulting in a gain of 8.57%. The small increase in reliability observed in their study was explained by the authors as being due to the fact that, in their dataset, only phenotypes of genotyped horses were available in the reference population. However, the same study reported that the increase in reliability was greater for genotyped animals and for animals with a small number of offspring. Furthermore, the authors demonstrated that the advantage of including genotype information would likely be more significant if the dataset included horses that were genotyped but not phenotyped. In a previous study, Haberland et al. [24] observed that the additional increase in accuracy obtained from GEBVs is small compared to traditional EBVs for animals with a large number of progeny records available, which is consistent with our results.

In horses, the generation interval is even longer (~10 years) [47] than it used to be in dairy cattle breeding before the implementation of genomic selection, and as a result, the reliability of breeding values is lower [29]. Even more, since some of the traits evaluated in the breed, such as functionality, rely on the full development of the individual to obtain reliable phenotypic values, phenotyping is delayed. In some breeds, this interval could be reduced through the use of reproductive biotechnologies, such as large embryo transfer programs or the extensive use of artificial insemination (AI) from young stallions, but this is not the case for the PRE breed. Our results support the idea that ssGBLUP could increase the reliability of individuals lacking phenotypic data, allowing their use as stallions or broodmares at early ages, thus reducing the generational interval and increasing genetic progress.

It is well established that the reliability of genomic predictions depends on the size and genetic composition of the reference population [48,49]. In cattle, the availability of extensive reference populations, often including millions of genotyped animals [50], has enabled substantial advancements in genomic selection. In contrast, systematic genotyping programs in horses are still underdeveloped. This creates an increasing imbalance, where the number of non-genotyped animals included in breeding programs is greater than that of genotyped ones. This disparity is likely to persist in the near future, even in populations actively pursuing genomic selection.

In this context, the single-step genomic prediction methodology is a particularly relevant approach in horse breeding. This method allows non-genotyped animals to contribute directly to the estimation system by integrating phenotypic, pedigree, and genotypic information into a unified framework [25,29]. Additionally, this method is especially interesting for breeds such as the Pura Raza Española (PRE) horse, which is in the initial phase of genomic selection, where genotypic data are still limited. In the PRE, the development of this approach within the breeding program can facilitate the implementation of genomic selection in equine populations, bridging the gap between genotyped and non-genotyped individuals and driving the genetic progress of the breed.

## 4. Conclusions

This study represents the first application of genomic evaluation using a single-step approach with a combined relationship matrix to assess morphology in the Pura Raza Española (PRE) horse. Our findings showed that the ssGREML method provided higher reliability for genomic estimated breeding values (GEBVs) compared to the traditional REML approach. However, this improvement could be further increased by expanding the number of genotyped animals in the reference population. These results indicate that the ssGREML method is a valid alternative to REML for the PRE breeding program, offering enhanced genetic gain, a shorter generation interval, and a more accurate selection of genetically superior animals. Future studies should focus on expanding the size of the reference population to further improve the reliability of genomic evaluations and maximize the potential benefits for this breed.

## Figures and Tables

**Table 1:** Descriptive statistics of morphological traits in the Pura Raza Española horse breed.

8	Trait	N	Mean ± SD	Min	Max	CV (%)
Zoometric traits, cm	HW	6861	161.84 ± 4.92	147	179	3.04
	SiL	7149	161.97 ± 5.29	144	183	3.27
	LS	7152	65.94 ± 3.97	43	81	6.03
	DsD	7149	74.01 ± 4.18	40	92	5.65
	LB	7151	30.39 ± 4.64	18	50	15.27
	WC	7087	53.47 ± 3.31	32	68	6.19
	LC	7149	52.82 ± 3.12	35	68	5.91
	TP	7141	191.59 ± 9.34	157	224	4.87
	PACB	7152	20.46 ± 1.26	15	33	6.18
	AS	6942	55.39 ± 6.68	2	82	12.05
	AC	6455	20.93 ± 5.67	2	59	27.10
Linear traits, class	LHA	7151	5.14 ± 0.95	1	9	18.39
	DHRV	7151	4.22 ± 0.91	1	7	21.53
Defect trait, class	CN	7118	1.316 ± 0.84	1	8	63.58

N: number of records; SD: standard deviation; CV: coefficient of variation; HW: height at withers; SiL: scapular-ischial length; LS: length of shoulder; DsD: dorsal-sternal diameter; LB: length of back; WC: width of croup; LC: length of croup; TP: thoracic perimeter; PACB: perimeter of anterior cannon bone; AS: angle of shoulder; AC: angle of croup; LHA: lateral hock angle; DHRV: direction of hock rear view; CN: cresty neck.

**Table 2:** Estimates of variance components and heritabilities obtained from the REML and ssGREML methods for morphological traits in the Pura Raza Española horse breed.

	REML			ssGREML		
Trait	$\sigma_{a}^{2}$	$\sigma_{e}^{2}$	$h^{2}$ (SE)	$\sigma_{a}^{2}$	$\sigma_{e}^{2}$	$h^{2}$ (SE)
HW	16.70	5.29	0.76 (0.038)	17.15	5.39	0.76 (0.033)
SiL	18.34	9.07	0.67 (0.039)	18.35	9.64	0.66 (0.036)
LS	8.81	7.14	0.55 (0.038)	8.37	7.79	0.52 (0.035)
DsD	7.84	9.72	0.45 (0.041)	7.53	10.13	0.43 (0.037)
LB	13.57	8.03	0.63 (0.037)	12.04	9.91	0.55 (0.036)
WC	5.49	5.08	0.52 (0.043)	5.32	5.35	0.50 (0.04)
LC	3.86	5.58	0.41 (0.036)	3.77	5.73	0.40 (0.034)
TP	47.48	35.45	0.57 (0.042)	44.49	39.04	0.53 (0.038)
PACB	0.45	0.91	0.33 (0.035)	0.45	0.92	0.33 (0.034)
AS	11.57	30.74	0.27 (0.033)	9.85	32.52	0.23 (0.03)
AC	9.09	22.83	0.28 (0.037)	8.75	23.39	0.27 (0.035)
LHA	0.22	0.66	0.25 (0.032)	0.20	0.68	0.23 (0.029)
DHRV	0.42	0.45	0.48 (0.04)	0.35	0.52	0.40 (0.036)
CN	0.06	0.63	0.08 (0.02)	0.06	0.62	0.09 (0.021)

$\sigma_{a}^{2}$: additive genetic variance; $\sigma_{e}^{2}$: residual variance; $h^{2}$: heritability; SE: standard error; HW: height at withers; SiL: scapular-ischial length; LS: length of shoulder; DsD: dorsal-sternal diameter; LB: length of back; WC: width of croup; LC: length of croup; TP: thoracic perimeter; PACB: perimeter of anterior cannon bone; AS: angle of shoulder; AC: angle of croup; LHA: lateral hock angle; DHRV: direction of hock rear view; CN: cresty neck.

**Table 3:** Comparison between the reliability obtained from the REML and ssGREML methods for morphological traits in the Pura Raza Española horse breed.

Trait	$R^{2}$ REML	$R^{2}$ ssGREML	% Increase in Reliability
HW	0.298	0.327	9.73
SiL	0.289	0.317	9.69
LS	0.272	0.293	7.72
DsD	0.247	0.272	10.12
LB	0.282	0.289	2.48
WC	0.265	0.288	8.68
LC	0.267	0.286	7.12
TP	0.268	0.293	9.33
PACB	0.250	0.274	9.60
AS	0.205	0.218	6.34
AC	0.203	0.230	13.30
LHA	0.209	0.224	7.18
DHRV	0.256	0.260	1.56
CN	0.176	0.185	5.11

$R^{2}$: reliability; HW: height at withers; SiL: scapular-ischial length; LS: length of shoulder; DsD: dorsal-sternal diameter; LB: length of back; WC: width of croup; LC: length of croup; TP: thoracic perimeter; PACB: perimeter of anterior cannon bone; AS: angle of shoulder; AC: angle of croup; LHA: lateral hock angle; DHRV: direction of hock rear view; CN: cresty neck.

**Table 4:** Comparison between the reliability obtained from the REML and ssGREML methods for morphology based on different criteria in the Pura Raza Española horse breed.

	Criteria							
	Sex		Number of Stallions’ Foals		Genotyped		Reliability	
	Stallions	Mares	≥40	<40	No	Yes	≥0.6	<0.6
HW	9.89	9.68	4.34	12.50	8.51	16.76	−0.32	32.60
SiL	9.06	9.67	3.87	12.50	8.03	16.33	0.09	32.20
LS	7.66	7.47	2.73	11.24	6.59	14.38	−0.75	29.33
DsD	10.04	10.24	3.50	14.04	8.94	17.79	0.44	18.78
LB	2.96	2.05	0.14	6.59	1.87	8.01	−5.91	24.40
WC	8.63	8.39	3.21	12.76	7.97	15.93	0.74	30.69
LC	7.03	6.88	2.36	11.52	6.32	13.57	−1.27	28.95
TP	8.91	9.03	3.18	12.60	8.27	16.38	1.15	31.19
PACB	10.00	10.12	3.88	14.04	8.86	17.06	1.88	29.70
AS	5.94	6.73	−0.35	11.64	5.61	12.99	−2.16	14.47
AC	13.00	13.59	5.08	19.25	12.37	22.70	−2.92	23.59
LHA	7.35	7.55	0.87	13.09	6.53	13.86	−1.75	15.09
DHRV	2.02	1.14	−1.49	5.96	0.82	6.93	−0.09	35.34
CN	5.20	4.47	−1.66	12.03	4.14	9.32	−0.21	10.39

HW: height at withers; SiL: scapular-ischial length; LS: length of shoulder; DsD: dorsal-sternal diameter; LB: length of back; WC: width of croup; LC: length of croup; TP: thoracic perimeter; PACB: perimeter of anterior cannon bone; AS: angle of shoulder; AC: angle of croup; LHA: lateral hock angle; DHRV: direction of hock rear view; CN: cresty neck.

## Data Availability

The data that support the findings of this study are property of the Royal National Association of Spanish Horse Breeders (ANCCE). Access to the data for scientific purposes could be requested directly from the breeders association (mejoragenetica@lgancce.es).

## References

[1] Ministerio de Agricultura, P.y.A.M (2023). Datos Censales (PURA RAZA ESPAÑOLA). https://servicio.mapa.gob.es/arca/flujos.html?_flowId=datosCensalesRaza-flow&tipoOperacion=CONSULTA&formatoPagina=0&id=50157.

[2] ANCCE Purebred Spanish Horse Breeding Program. https://www.lgancce.com/Documentacion/Normativa/Nacional/programa_cria_en.pdf.

[3] Sánchez-Guerrero M.J., Molina A., Gómez M.D., Peña F., Valera M. (2016). Relationship between morphology and performance: Signature of mass-selection in Pura Raza Español horse. Livest. Sci..

[4] ANCCE New Assessment Record Sheet for Basic Approval as a Breeding Stock and Genetic Assessment for Morphology. https://www.lgancce.com/web/news/new-assessment-record-sheet-basic-approval-breeding-stock-and-genetic-assessment-morphology?lang=en.

[5] Blasco A. (2022). Animal breeding methods and sustainability. Animal Breeding and Genetics.

[6] Meuwissen T.H., Hayes B.J., Goddard M.E. (2001). Prediction of total genetic value using genome-wide dense marker maps. Genetics.

[7] Aguilar I., Misztal I., Johnson D.L., Legarra A., Tsuruta S., Lawlor T.J. (2010). Hot topic: A unified approach to utilize phenotypic, full pedigree, and genomic information for genetic evaluation of Holstein final score. J. Dairy Sci..

[8] Christensen O.F., Lund M.S. (2010). Genomic prediction when some animals are not genotyped. Genet. Sel. Evol..

[9] Legarra A., Christensen O.F., Aguilar I., Misztal I. (2014). Single Step, a general approach for genomic selection. Livest. Sci..

[10] Lourenco D., Legarra A., Tsuruta S., Masuda Y., Aguilar I., Misztal I. (2020). Single-Step Genomic Evaluations from Theory to Practice: Using SNP Chips and Sequence Data in BLUPF90. Genes.

[11] Koivula M., Strandén I., Aamand G.P., Mäntysaari E.A. (2016). Effect of cow reference group on validation reliability of genomic evaluation. Animal.

[12] Koivula M., Stranden I., Su G., Mantysaari E.A. (2012). Different methods to calculate genomic predictions--comparisons of BLUP at the single nucleotide polymorphism level (SNP-BLUP), BLUP at the individual level (G-BLUP), and the one-step approach (H-BLUP). J. Dairy Sci..

[13] Mantysaari E.A., Koivula M., Stranden I. (2020). Symposium review: Single-step genomic evaluations in dairy cattle. J. Dairy Sci..

[14] Oliveira H.R., Lourenco D.A.L., Masuda Y., Misztal I., Tsuruta S., Jamrozik J., Brito L.F., Silva F.F., Schenkel F.S. (2019). Application of single-step genomic evaluation using multiple-trait random regression test-day models in dairy cattle. J. Dairy Sci..

[15] Massender E., Brito L.F., Maignel L., Oliveira H.R., Jafarikia M., Baes C.F., Sullivan B., Schenkel F.S. (2022). Single-and multiple-breed genomic evaluations for conformation traits in Canadian Alpine and Saanen dairy goats. J. Dairy Sci..

[16] Mucha S., Mrode R., MacLaren-Lee I., Coffey M., Conington J. (2015). Estimation of genomic breeding values for milk yield in UK dairy goats. J. Dairy Sci..

[17] Teissier M., Larroque H., Robert-Granie C. (2019). Accuracy of genomic evaluation with weighted single-step genomic best linear unbiased prediction for milk production traits, udder type traits, and somatic cell scores in French dairy goats. J. Dairy Sci..

[18] Baloche G., Legarra A., Salle G., Larroque H., Astruc J.M., Robert-Granie C., Barillet F. (2014). Assessment of accuracy of genomic prediction for French Lacaune dairy sheep. J. Dairy Sci..

[19] Li L., Gurman P.M., Swan A.A., Brown D.J. (2021). Single-step genomic evaluation of lambing ease in Australian terminal sire breed sheep. Anim. Prod. Sci..

[20] Wei C., Luo H., Zhao B., Tian K., Huang X., Wang Y., Fu X., Tian Y., Di J., Xu X. (2020). The Effect of Integrating Genomic Information into Genetic Evaluations of Chinese Merino Sheep. Animals.

[21] Fu C., Ostersen T., Christensen O.F., Xiang T. (2021). Single-step genomic evaluation with metafounders for feed conversion ratio and average daily gain in Danish Landrace and Yorkshire pigs. Genet. Sel. Evol..

[22] Song H., Zhang J., Zhang Q., Ding X. (2018). Using Different Single-Step Strategies to Improve the Efficiency of Genomic Prediction on Body Measurement Traits in Pig. Front. Genet..

[23] Xiang T., Nielsen B., Su G., Legarra A., Christensen O.F. (2016). Application of single-step genomic evaluation for crossbred performance in pig. J. Anim. Sci..

[24] Haberland A.M., Konig von Borstel U., Simianer H., Konig S. (2012). Integration of genomic information into sport horse breeding programs for optimization of accuracy of selection. Animal.

[25] Mark T., Jönsson L., Holm M., Christiansen K. Towards genomic selection in Danish Warmblood horses: Expected impacts and selective genotyping strategy. Proceedings of the 10th World Congress on Genetics Applied to Livestock Production.

[26] Vosgerau S., Krattenmacher N., Falker-Gieske C., Seidel A., Tetens J., Stock K.F., Nolte W., Wobbe M., Blaj I., Reents R. (2022). Genetic and genomic characterization followed by single-step genomic evaluation of withers height in German Warmblood horses. J. Appl. Genet..

[27] Ricard A., Crevier-Denoix N., Pourcelot P., Crichan H., Sabbagh M., Dumont-Saint-Priest B., Danvy S. (2023). Genetic analysis of geometric morphometric 3D visuals of French jumping horses. Genet. Sel. Evol..

[28] Eggen A. (2012). The development and application of genomic selection as a new breeding paradigm. Anim. Front..

[29] Stock K.F., Jönsson L., Ricard A., Mark T. (2016). Genomic applications in horse breeding. Anim. Front..

[30] Sánchez M.J., Gómez M.D., Molina A., Valera M. (2013). Genetic analyses for linear conformation traits in Pura Raza Español horses. Livest. Sci..

[31] Carter R.A., Geor R.J., Burton Staniar W., Cubitt T.A., Harris P.A. (2009). Apparent adiposity assessed by standardised scoring systems and morphometric measurements in horses and ponies. Vet. J..

[32] R-Core-Team (2024). R: A Language and Environment for Statistical Computing V4.4.2 “Pile of Leaves”.

[33] Purcell S., Neale B., Todd-Brown K., Thomas L., Ferreira M.A., Bender D., Maller J., Sklar P., de Bakker P.I., Daly M.J. (2007). PLINK: A tool set for whole-genome association and population-based linkage analyses. Am. J. Hum. Genet..

[34] Marschner I. (2011). GLM2: Fitting generalized linear models with convergence problems. R. J..

[35] VanRaden P.M. (2008). Efficient methods to compute genomic predictions. J. Dairy Sci..

[36] Yin L., Zhang H., Tang Z., Yin D., Fu Y., Yuan X., Li X., Liu X., Zhao S. (2023). HIBLUP: An integration of statistical models on the BLUP framework for efficient genetic evaluation using big genomic data. Nucleic Acids Res..

[37] Poyato-Bonilla J., Sanchez-Guerrero M.J., Cervantes I., Gutierrez J.P., Valera M. (2021). Genetic parameters for canalization analysis of morphological traits in the Pura Raza Espanol horse. J. Anim. Breed. Genet..

[38] Sánchez M.J., Azor P.J., Molina A., Parkin T., Rivero J.L., Valera M. (2017). Prevalence, risk factors and genetic parameters of cresty neck in Pura Raza Español horses. Equine Vet. J..

[39] Poyato-Bonilla J., Perdomo-Gonzalez D.I., Sanchez-Guerrero M.J., Varona L., Molina A., Casellas J., Valera M. (2020). Genetic inbreeding depression load for morphological traits and defects in the Pura Raza Espanola horse. Genet. Sel. Evol..

[40] Clement V., Bibe B., Verrier E., Elsen J.M., Manfredi E., Bouix J., Hanocq E. (2001). Simulation analysis to test the influence of model adequacy and data structure on the estimation of genetic parameters for traits with direct and maternal effects. Genet. Sel. Evol..

[41] Christensen O.F., Madsen P., Nielsen B., Ostersen T., Su G. (2012). Single-step methods for genomic evaluation in pigs. Animal.

[42] Ziadi C., Perdomo-González D., Valera M., Laseca N., Encina A., Azor P., Rodríguez A., Demyda-Peyrás S., Molina A. Genomics improves the reliability of Breeding Value Prediction of morphological and reproductive traits in the Pura Raza Español Horse: Preliminary results. Proceedings of the 46th ICAR Annual Conference.

[43] VanRaden P.M., Tooker M.E., Wright J.R., Sun C., Hutchison J.L. (2014). Comparison of single-trait to multi-trait national evaluations for yield, health, and fertility. J. Dairy Sci..

[44] Misztal I., Lourenco D., Legarra A. (2020). Current status of genomic evaluation. J. Anim. Sci..

[45] Guarini A.R., Lourenco D.A.L., Brito L.F., Sargolzaei M., Baes C.F., Miglior F., Misztal I., Schenkel F.S. (2018). Comparison of genomic predictions for lowly heritable traits using multi-step and single-step genomic best linear unbiased predictor in Holstein cattle. J. Dairy Sci..

[46] Gao H., Christensen O.F., Madsen P., Nielsen U.S., Zhang Y., Lund M.S., Su G. (2012). Comparison on genomic predictions using three GBLUP methods and two single-step blending methods in the Nordic Holstein population. Genet. Sel. Evol..

[47] Valera M., Molina A., Gutiérrez J.P., Gómez J., Goyache F. (2005). Pedigree analysis in the Andalusian horse: Population structure, genetic variability and influence of the Carthusian strain. Livest. Prod. Sci..

[48] Goddard M. (2009). Genomic selection: Prediction of accuracy and maximisation of long term response. Genetica.

[49] van den Berg I., Meuwissen T.H.E., MacLeod I.M., Goddard M.E. (2019). Predicting the effect of reference population on the accuracy of within, across, and multibreed genomic prediction. J. Dairy Sci..

[50] Lozada-Soto E.A., Tiezzi F., Jiang J., Cole J.B., VanRaden P.M., Maltecca C. (2022). Genomic characterization of autozygosity and recent inbreeding trends in all major breeds of US dairy cattle. J. Dairy Sci..

